# Intestinal organoids in farm animals

**DOI:** 10.1186/s13567-021-00909-x

**Published:** 2021-02-25

**Authors:** Martin Beaumont, Fany Blanc, Claire Cherbuy, Giorgia Egidy, Elisabetta Giuffra, Sonia Lacroix-Lamandé, Agnès Wiedemann

**Affiliations:** 1 GenPhySE, Université de Toulouse, INRAE, ENVT, Castanet-Tolosan, 31326 France; 2 GABI, INRAE, AgroParisTech, Université Paris‐Saclay, Jouy‐en‐Josas, 78350 France; 3 Micalis, INRAE, AgroParisTech, Université Paris-Saclay, Jouy-en-Josas, 78350 France; 4 ISP, INRAE, Université de Tours, Nouzilly, 37380 France; 5 IRSD - Institut de Recherche en Santé Digestive, Université de Toulouse, INSERM, INRAE, ENVT, UPS, Toulouse, France

**Keywords:** Enteroids, Epithelium, Gut, Monolayer, Culture, Polarity, Pig, Bovine, Chicken, Horse, Rabbit

## Abstract

In livestock species, the monolayer of epithelial cells covering the digestive mucosa plays an essential role for nutrition and gut barrier function. However, research on farm animal intestinal epithelium has been hampered by the lack of appropriate in vitro models. Over the past decade, methods to culture livestock intestinal organoids have been developed in pig, bovine, rabbit, horse, sheep and chicken. Gut organoids from farm animals are obtained by seeding tissue-derived intestinal epithelial stem cells in a 3-dimensional culture environment reproducing in vitro the stem cell niche. These organoids can be generated rapidly within days and are formed by a monolayer of polarized epithelial cells containing the diverse differentiated epithelial progeny, recapitulating the original structure and function of the native epithelium. The phenotype of intestinal organoids is stable in long-term culture and reflects characteristics of the digestive segment of origin. Farm animal intestinal organoids can be amplified in vitro, cryopreserved and used for multiple experiments, allowing an efficient reduction of the use of live animals for experimentation. Most of the studies using livestock intestinal organoids were used to investigate host-microbe interactions at the epithelial surface, mainly focused on enteric infections with viruses, bacteria or parasites. Numerous other applications of farm animal intestinal organoids include studies on nutrient absorption, genome editing and bioactive compounds screening relevant for agricultural, veterinary and biomedical sciences. Further improvements of the methods used to culture intestinal organoids from farm animals are required to replicate more closely the intestinal tissue complexity, including the presence of non-epithelial cell types and of the gut microbiota. Harmonization of the methods used to culture livestock intestinal organoids will also be required to increase the reproducibility of the results obtained in these models. In this review, we summarize the methods used to generate and cryopreserve intestinal organoids in farm animals, present their phenotypes and discuss current and future applications of this innovative culture system of the digestive epithelium.

## Introduction

**Figure 1:**
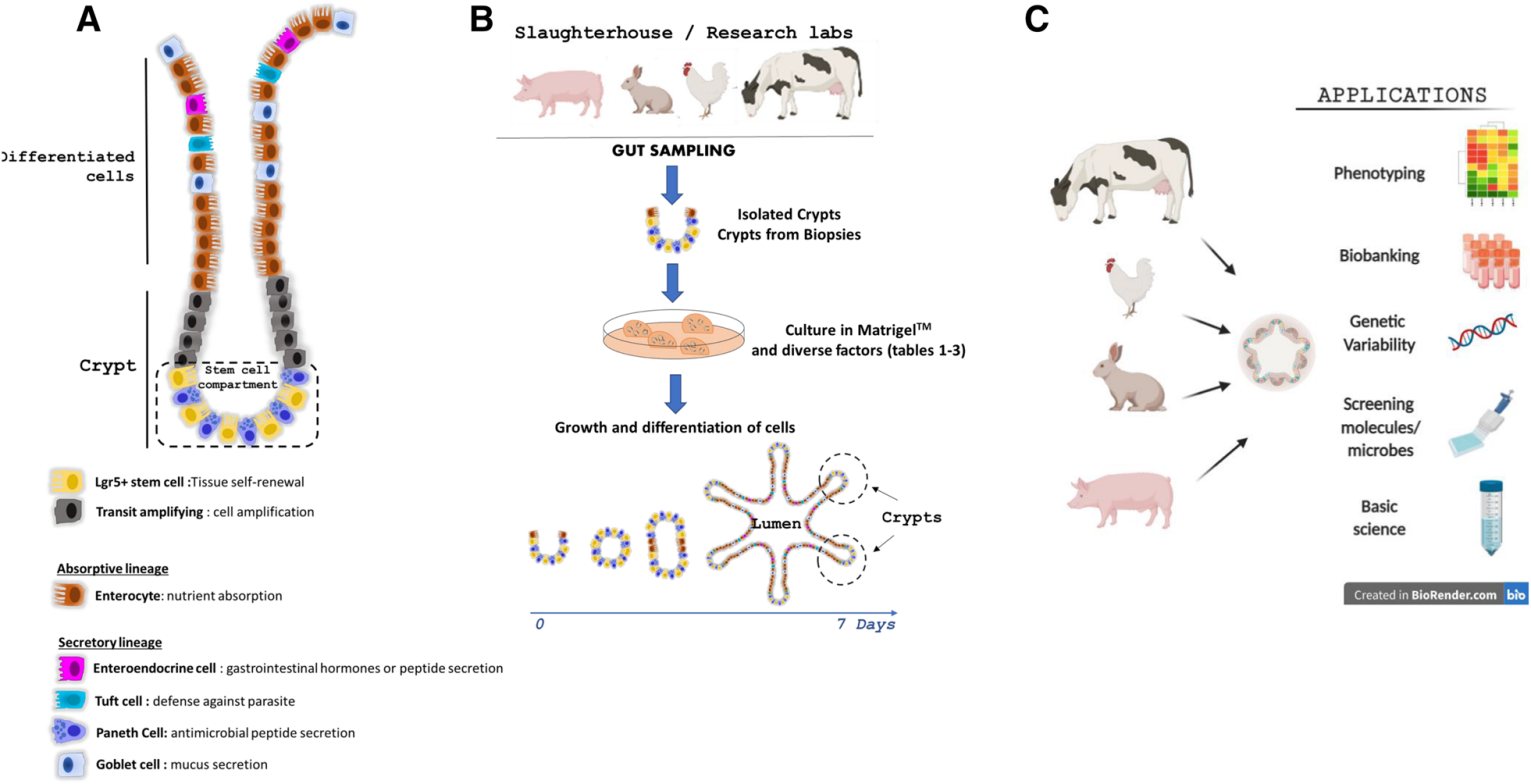
**Models of intestinal organoids in farm animals**. **A** Diverse cell lineages constitute the digestive epithelium. Paneth and stem cells are localized at the bottom of the crypts, while enterocytes, goblet cells, enteroendocrine cells and tuft cells migrate towards the lumen upon differentiation. **B** Schematic illustration of livestock intestinal organoid generation from epithelial crypts isolated from fresh intestinal tissue or from frozen biopsies. **C** Schematic representation of farm animal intestinal organoid applications in basic and applied science.

The intestinal epithelium is formed by a monolayer of cells located at the mucosal surface. Intestinal epithelial cells (IEC) contribute to food digestion and nutrient absorption while acting as a physical and immunological barrier against harmful luminal components (microorganisms, toxins, food antigens) [1]. This dual function of the intestinal epithelium is performed by the coordinated action of differentiated epithelial cells specialized for nutrient absorption (enterocytes), hormone secretion (enteroendocrine cells), antimicrobial peptide secretion (Paneth cells), anti-parasite immunity (tuft cells) or mucus secretion (goblet cells) (Figure 1A). All these differentiated IEC types derive from intestinal epithelial stem cells (IESC) located at the base of epithelial crypts [2]. Paneth cells remain at the bottom of crypts whereas the other IEC types differentiate while they migrate towards the lumen. Epithelial cell type distribution also differs along the length of the intestine, which reflects the functional specialization of digestive segments [3]. The small intestine is divided into 3 segments: duodenum, jejunum and ileum. The duodenum receives the food chyme, bile juice and pancreatic secretions to complete the chemical digestion. The jejunum is the main site for nutrient absorption while the ileum absorbs residual nutrients, vitamins and conjugated bile acids. The large intestine is composed of the caecum and the colon, which main functions are to absorb water, electrolytes and microbial fermentation products.

The study of the intestinal epithelium in livestock species has major implications for agricultural (e.g. improvement of feed efficiency), veterinary (e.g. resistance to enteric pathogens) or biomedical (e.g. large animal models of human diseases) research. However, models commonly used to study livestock epithelium present major limitations. In vitro immortalized intestinal epithelial cell lines (e.g. porcine IPEC-J2) lack cellular heterogeneity, do not fully reproduce tissue functionality and present genomic abnormalities [4, 5] or have not been established for some species (e.g. chicken, bovine). Ex vivo culture of intestinal explants or primary isolated IECs recapitulate key features of the in vivo tissue but they are not suitable for long-term experiments due to limited viability (24–48 h as reviewed by Randall et al. [6]). The recent development of livestock intestinal organoids solved most of these limitations.

Intestinal organoids are self-organized 3D structures composed of a monolayer of polarized IEC surrounding a hollow lumen [7] (Figure 1B). This innovative model recapitulates in vitro the multicellular composition of the intestinal epithelium, its architecture with crypt-domains and its main roles such as nutrient absorption and barrier function. “Enteroids” and “colonoids” refer to intestinal organoids derived from the small intestine or from the colon, respectively [8]. Importantly, intestinal organoids retain in vitro phenotypic features of their digestive segment of origin [3]. Intestinal organoid culture was first developed in mouse and humans after identification of the signaling pathways involved in the maintenance and proliferation of Lgr5^+^ IESC [9]. Intestinal organoid culture is based on the reproduction of the IESC niche in vitro: Wnt pathway activation, epidermal growth factor (EGF) signaling stimulation and bone morphogenic protein (BMP) pathway inhibition [2]. IESC are seeded in a gel of extracellular matrix proteins (e.g. Matrigel™) that provides a structural scaffold for 3D growth and promotes cell survival (Figure 1B). Human and mouse intestinal organoids can be derived from intestinal tissue-derived IESC or from induced pluripotent stem (iPS) or embryonic stem (ES) cells [10]. Organoids obtained from tissue-derived IESC are composed only of epithelial cells. In contrast, both epithelial and mesenchymal cells (e.g. myofibroblasts) are present in iPS and ES-derived intestinal organoids, which however remain in an immature, fetal-like state [10].

Herein, we first review the main methods used to generate and cryopreserve intestinal organoids from farm animals. Then, we present the main characteristics of these innovative intestinal epithelium culture systems. Finally, we detail current and future applications of livestock intestinal organoids (Figure 1C).

## Methods to generate and cryopreserve livestock intestinal organoids

### Methods to grow intestinal organoids

**Figure 2:**
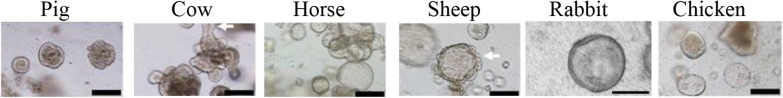
**Morphology of intestinal organoids from farm animals**. Brightfield images of intestinal organoids grown in Matrigel™ with 50% L-WRN conditioned media. Organoids from pig, cow, horse and sheep were obtained from the terminal ileum. Organoids from rabbit and chicken were obtained from the caecum. Scale bars: 200 µM. Images were adapted from previous publications distributed under the terms of Creative Commons Licenses: Powell and Behnke (pig, cow, horse, sheep and chicken organoids) [14] and from Mussard et al. (rabbit organoids) [16].

Despite recent progress for the development of domestic animal iPS and ES cells [11, 12], to our knowledge, all intestinal organoid models from livestock species have been developed with tissue-derived-IESC. Methods originally developed to culture mouse and human intestinal organoids from tissue-derived IESC [13, 14] were recently adapted to numerous farm animal species including pig, rabbit, cow, sheep, horse and chicken (summarized in Table 1 for pig, Table 2 for ruminant and herbivorous species and Table 3 for chicken) (Figure 2). Farm animal intestinal organoids were successfully obtained from several digestive segments (duodenum, jejunum, ileum, caecum and colon). The first step of livestock intestinal organoid culture is to isolate epithelial crypts that contain IESC. Most of the studies used a dissociation buffer containing ethylenediaminetetraacetic acid (EDTA) and dithiothreitol (DTT). There are great variations in EDTA concentration (0.8–30 mM), time and temperature of incubation according to the studies, species and digestive segments (Tables 1, 2 and 3). Some studies supplemented the crypt isolation buffer with Y27632, a ROCK inhibitor that prevents epithelial cell death. Isolated crypts are then seeded in Matrigel™ and the growth medium is added. Alternatively, a hanging drop culture system without Matrigel™ embedding has been reported for embryonic chicken intestinal organoids [15].

**Table 1:** Pig intestinal organoids

Segment	Application	Crypt isolation	Growth medium	Reference
Duodenum	Lipopolysaccharides challenge	30 mM EDTA, 1.5 mM DTT, 4 °C, 20 min + 30 mM EDTA, 10 µM Y27632, warm, 10 min	DMEM, N2, B27 without vitamin A, 10 mM HEPES, 2 mM L-glutamine, 1 μg/mL R-spondin1, 100 ng/mL Noggin, 50 ng/mL EGF, 2.5 ng/mL Wnt-3a	[60]
	Gene edited pigs	1 mg/mL collagenase, 2 mg/mL BSA, 37 °C, 30 min	IntestiCult™ (STEMCELL Technologies, Vancouver, Canada)	[37]
Jejunnum	Cell lineage identification	30 mM EDTA, 1 mM DTT, 10 µM Y27632, 4 °C, 20 min and 37 °C 10 min	DMEM/F12, N-2, B-27 minus vitamin A, Glutamax, 1 mM HEPES, 1 mM NAC, 100 ng/mL rm Noggin, 500 ng/mL rh R-spondin, 50 ng/mL rm EGF, 100 ng/mL rh Wnt3a, 10 µM Y-27632, 10 µM SB202190, 500 nM LY2157299	[39]
	Lentivirus transduction	10 mM EDTA, 1 mM DTT, 4 °C, 30 min	DMEM/F-12, 2 mM glutaMAX, 10 mM HEPES, 1 mM NAC, N2, B27, 50 ng/mL rm EGF, 100 ng/mL rm Noggin, 500 ng/mL rh R-spondin 1, Wnt3a CM, 1 mM valproic acid, 10 mM nicotinamide, 2.5 μM CHIR99021, 10 μM Y27632, 10 μM SB202190, 500 nM LY2157299	[25]
	2D monolayers, transcriptomics	2 mM EDTA, 4 °C, 30 min	DMEM/F12, 10 mM HEPES, B-27, 1.25 mM NAC, 50 ng/mL rh EGF, 15 nM gastrin, 10 mM nicotinamide, 10 μM p38 MAPK inhibitor, 600 nM A83-01, Noggin CM (15% v/v), R-spondin CM (15% v/v), and Wnt3A CM (30% v/v)	[5, 19]
	Mycotoxin challenge	30 mM EDTA, 1.5 mM DTT, 10 µM Y27632, 4 °C, 30 min	DMEM, 10% FBS, N2, B27, 1X GlutaMAX, 1 mM NAC, 50 ng/mL EGF, 100 ng/mL Noggin, 500 ng/mL R-spondin1, 10 mM nicotinamide, 10 µM SB202190, 0.5 µM LY2157299, 45% Wnt3a CM, 10 µM Y27632 (2–3 first days)	[18]
	Heat Stress	30 mM EDTA, 1.5 mM DTT, 4 °C, 30 min	DMEM/F12, Wnt3a CM, rm EGF, rm Noggin, rh R-spondin1, nicotinamide, Y27632, LY2157299, SB202190, CHIR99021, FBS, N2, B27, glutamine, NAC	[26]
	Swine enteric virus infection	5 mM EDTA, 4 °C, 1 h	IntestiCult™ (STEMCELL Technologies, Vancouver, Canada)	[34]
	*S.* Typhimurium and *T. gondii* infection	0.8 mM EDTA, 4 °C, 30 min	IntestiCult™ (STEMCELL Technologies, Vancouver, Canada)	[20]
Ileum	Model development	Collagenase, 37 °C, 1.5 h	50% (DMEM + 10% FBS), 50% L-WRN CM, 10 µM SB431542, 10 µM Y27632	[14]
	Vitamin A	2 mM EDTA, 4 °C, 30 min	Wnt3a, Noggin and R-spondin 1 CM, B27, N2, glutamine, NAC, rm EGF, nicotinamide, SB202190	[45]
	Mucus production	30 mM EDTA, 1 mM DTT, 37 °C, 10–15 min	IntestiCult™ (STEMCELL Technologies, Vancouver, Canada)	[43]
	*Lawsonia intracellularis* infection	30 mM EDTA, 1 mM DTT, 37 °C, 5–15 min	IntestiCult™ (STEMCELL Technologies, Vancouver, Canada)	[36]
Duodenum, Jejunum, Ileum, Colon	Porcine epidemic diarrhea virus infection	Cell dissociation reagent (STEMCELL Technologies,Vancouver, Canada)	IntestiCult™ (STEMCELL Technologies, Vancouver, Canada)	[21]

CM: conditioned medium, NAC: N-acetyl cysteine, L-WRN: L cell line engineered to secrete Wnt3a, R-spondin 3 and Noggin, rm: recombinant mouse, rh: recombinant human, FBS: fetal bovine serum, EGF: epidermal growth factor. Antibiotics and antifungals added in the growth medium are not presented.

**Table 2:** Ruminant and herbivorous species (bovine, sheep, horse and rabbit) intestinal organoids

Species	Segment	Application	Crypt isolation	Growth medium	Reference
Bovine	Jejunum	*S.* Typhimurium and *T. gondii* infection	0.8 mM EDTA, 4 °C, 30 min	50% IntestiCult™ (STEMCELL Technologies, Vancouver, Canada) medium, 50% Wnt3a CM, 1 μg/mL rh R-spondin, 100 ng/mL rm Noggin, 100 ng/mL rm EGF, 1.5 μM CHIR99021, 5 μM Y27632, 5 μM SB202190, 250 nM A8301	[20]
	Ileum	Model development	1% FBS, 75 U/mL collagenase 1-A, 20 µg/mL dispase I, 37 °C, 40 min	IntestiCult™ (mouse) (STEMCELL Technologies, Vancouver, Canada), Y27632, LY2157299, SB202190	[22]
		*E. coli* infection	1% FBS, 75 U/mL collagenase 1-A, 20 µg/mL dispase I, 37 °C, 40 min	IntestiCult™ (mouse) (STEMCELL Technologies, Vancouver, Canada), 10 µM Y27632, 500 nM LY2157299, 10 µM SB202190	[47]
		Rotavirus infection	Not described	DMEM/F12, 10 mM HEPES, GlutaMAX, 50 ng/mL EGF, 10% Noggin CM, 20% R-spondin CM, 50% Wnt3a CM, 10 mM nicotinamide, 10 nM gastrin I, 500 nM A-83–01, 10 μM SB202190, B27, N2, 1 mM NAC	[24]
		Model development	Collagenase, 37 °C, 1.5 h	50% (DMEM + 10% FBS), 50% L-WRN CM, 10 µM SB431542, 10 µM Y27632	[14]
	Colon	2D monolayer, in-plate cryoconservation	Not described	DMEM/F12, 1% BSA, 2 mM Glutamine, N2, B27, 10 mM HEPES, 50% Wnt3a-CM, 1 µg/mL rh R-spondin, 100 ng/mL rh Noggin, 50 ng/mL rh EGF, 500 nM A-83–01, 10 μM SB202190, 10 nM [Leu]15-gastrin-1, 10 mM nicotinamide, 1 mM NAC, 5 μM CHIR99021, 10 μM Y-27623, 50 nm Prostaglandin E2, 2 mM sodium acetate	[23]
Sheep	Ileum	Model development	Collagenase, 37 °C, 1.5 h	50% (DMEM + 10% FBS), 50% L-WRN CM, 10 µM SB431542, 10 µM Y27632	[14]
Horse	Ileum	Model development	Collagenase, 37 °C, 1.5 h	50% (DMEM + 10% FBS), 50% L-WRN CM, 10 µM SB431542, 10 µM Y27632	[14]
	Jejunum	Model development	30 mM EDTA, 10 mM Y27632, 1 mM DTT, 4 °C, 30 min + 30 mM EDTA, 10 mM Y27632, 37 °C, 10 min	DMEM/F12, N-2, B27, GlutaMAX, 1 mM Hepes, 100 ng/mL hr noggin, 500 ng/mL rh R-spondin, 50 ng/mL hr EGF, 100 ng/mL, hr Wnt3a, 10 mM Y27632, 10 mM SB202190, 500 nM LY215799, 2,5 µM CHIR99021	[27]
Rabbit	Caecum	Model development	9 mM EDTA, 3 mM DTT, 10 µM Y27632, room temperature, 30 min	Medium 1: DMEM, 10% FBS, 1 mM HEPES, 0.5 mM NAC, 10 µM CHIR99021, 10 µM Y27632,10 µM SB431542, 0.2 µM LDN193189 Medium 2: 50% (DMEM + 10% FBS), 50% L-WRN conditioned medium, 10 µM Y27632, 10 µM SB431542	[16]

CM: conditioned medium, NAC: N-acetyl cysteine, L-WRN: L cell line engineered to secrete Wnt3a, R-spondin 3 and Noggin, rm: recombinant mouse, rh: recombinant human, FBS: fetal bovine serum, EGF: epidermal growth factor. Antibiotics and antifungals added in the growth medium are not presented.

**Table 3:** Chicken intestinal organoids

Segment	Application	Segment	Crypt isolation	Growth medium	Reference
Caecum	Model development	Caecum	Collagenase, 37 °C, 1.5 h	50% (DMEM + 10% FBS), 50% L-WRN CM, 10 µM SB431542, 10 µM Y27632	[14]
Jejunum	Model development	Jejunum	2 mM EDTA, 4 °C, 3 × 30 min	DMEM/F12, 10 mM HEPES, 2 mM glutaMAX, 50 ng/mL EGF, 100 ng/mL Noggin, 500 ng/mL R-spondin1	[40]
Small intestine	Chemical treatments	Small intestine	Mechanical	DMEM/F12, 10% FBS, insulin-transferrin-selenium, polyamine, bovine pituitary extract	[61]
Embryonic Small intestine	Model development, TLR agonist and *Lactobacillus acidophilus* treatment	Embryonic Small intestine	2.5 mM EGTA, 0.5% glucose, 4 °C, 15 min + 45 min twice	DMEM/F12, Glutamax, insulin-transferrin-selenium premix, 25 ng/mL EGF, 25 ng/mL rh Noggin,250 ng/mL rh R-spondin1, 5 µg/mL Prostaglandin E2	[15, 31, 41, 42, 62]

CM: conditioned medium, L-WRN: L cell line engineered to secrete Wnt3a, R-spondin 3 and Noggin, rh: recombinant human, FBS: fetal bovine serum, EGF: epidermal growth factor. Antibiotics and antifungals added in the growth medium are not presented.

The composition of livestock intestinal organoid growth medium has been directly adapted from human and mouse protocols (see Tables 1, 2 and 3 for references). Although recombinant growth factors are most often not commercially available for farm animals, human or mouse orthologues can be used due to high evolutionary conservation of their amino acid sequences [14, 16]. Wnt signaling activation is generally induced by mouse or human recombinant Wnt3a and R-spondin, either used purified or in conditioned media from engineered cell lines. Some studies also used CHIR99021, an inhibitor of glycogen synthase kinase 3 to further activate the Wnt pathway. BMP pathway signaling is usually inhibited by human or mouse recombinant Noggin, either used purified or in conditioned media. For instance, the conditioned medium from L-WRN cells (mouse L cell line secreting Wnt3a, R-spondin and Noggin, ATCC® CRL-3276™) was successfully used to culture intestinal organoids from several species [14, 16] (Figure 2). The BMP inhibitor LDN193189 was used instead of Noggin in the growth medium of rabbit caecum organoids [16]. TGF-β receptor inhibitors (LY2157299, A8301, SB43542) and the p38 MAPK inhibitor SB202190 were used in several studies to promote epithelial proliferation and inhibit differentiation [17]. Epithelial proliferation is stimulated by human or murine EGF in several studies, while other methods used fetal bovine serum (FBS) that is a potent source of growth factors, though undefined. When organoid culture medium does not contain FBS, the cell culture supplements N2 and B27 are used to provide vitamins or hormones. Most studies supplemented the growth medium with the ROCK inhibitor Y27632 to prevent isolated epithelial cell death but it can be omitted after 2–3 days of culture when organoids are formed [18]. Continuous use of Y27632 was also reported to prevent the formation of tight junctions in pig intestinal organoid cells, when cultured in 2D monolayers (described below) [19]. Other common constituents of livestock intestinal organoid growth media include HEPES (buffer), N-acetylcysteine (antioxidant), GlutaMAX (stable glutamine), nicotinamide and antibiotics/antifungals. As an alternative to custom-made culture media, pig and bovine intestinal organoids can be cultured in IntestiCult™, a proprietary defined medium available from STEMCELL Technologies (Vancouver, Canada) [20–22] (Tables 1 and 2). This commercial growth medium has been optimized for mouse and human organoids but not for other species. Indeed, supplementation of mouse IntestiCult™ organoid growth medium with additional growth factor was required to culture bovine organoids [20, 22] (Table 2). After initial growth, intestinal organoid differentiation can be enhanced by reducing the concentration of the IESC niche factors. For instance, rabbit caecum organoids differentiation was obtained by reducing the concentration of L-WRN conditioned medium from 50 to 5% for 2 days [16]. Similarly, bovine enteroid differentiation was triggered by withdrawal of Wnt3a and other niche factors [23, 24].

Intestinal organoid culture medium is replaced every 2–3 days. After 5–10 days of culture, intestinal organoids are dissociated by mechanical and/or enzymatic methods and are subcultured with a 1:3 to 1:8 dilution ratio in fresh Matrigel™. A study showed that pig enteroids cultured at 39 °C (body temperature of pigs) expressed lower levels of the IESC marker Lgr5 ^+^, when compared to organoids grown at 37 °C [25]. This result suggests that the temperature of cell culture incubators should be adapted to each species. Another study in pig enteroids showed that the organoid forming efficiency was higher when jejunum crypts were cultured at 37 °C, when compared to 41 °C, an experimental condition used to mimic heat stress [26]. However, potentially lower stability of human and mouse recombinant growth factors at temperatures above 37 °C might contribute to the effects of temperature on organoid phenotype.

### Organoid cryopreservation and biobanking

**Figure 3:**
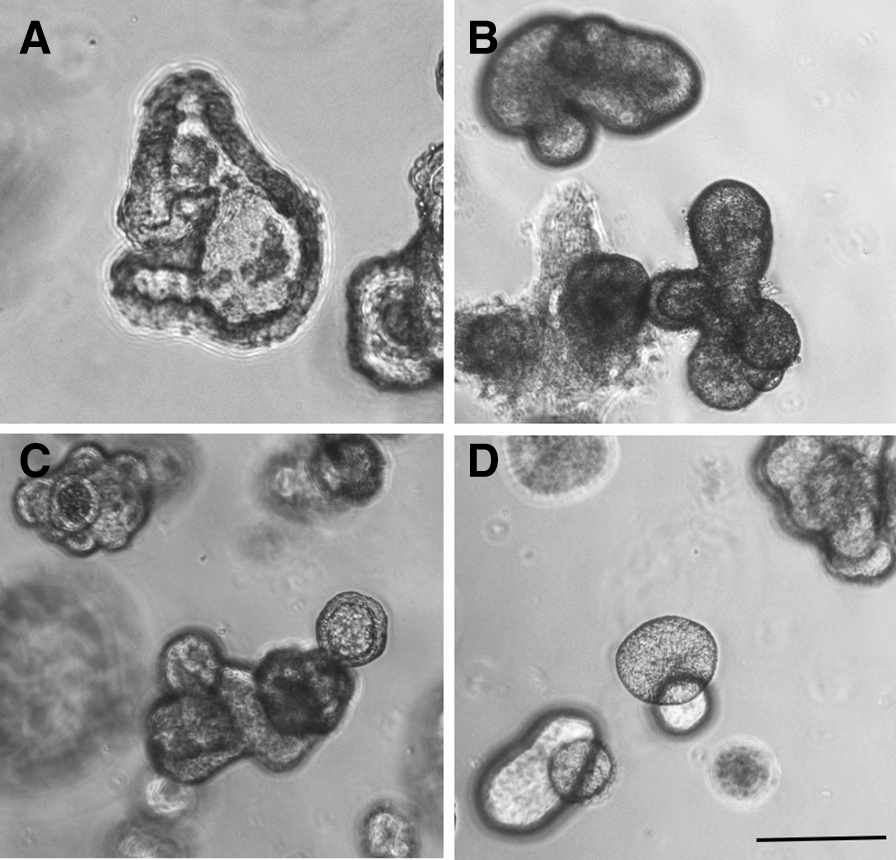
**Morphological features of porcine gut organoids obtained from frozen tissues**. Organoids are derived from: **A** duodenum (7 days, passage 3), **B** jejunum (7 days, passage 1), **C** ileum (7 days, passage 3) and **D** colon (8 days, passage 1). Observation by phase contrast microscopy. Bars: 200 µm. Images are representative of organoids obtained from 4 pigs for each digestive segment.

Farm animal intestinal organoids can be successfully recovered after cryopreservation in freezing medium (containing FBS, dimethyl sulfoxide and in some studies Y27632) and long-term storage in liquid nitrogen [14, 16, 20, 25]. High-throughput applications (e.g. bioactive molecules screening) might benefit from the recent development of an in-plate cryopreservation technique for bovine colonic organoids [23]. However, since slaughterhouses are often located away from the cell culture laboratory, the isolation and culture of intestinal epithelial crypts from fresh tissues is not always feasible for farm animals. Methods have been developed to directly cryopreserve either isolated epithelial crypts or intestinal tissues retaining the ability to generate organoids after thawing. These methods allow sampling of a large number of animals at once, which would not be compatible with immediate labor-intensive organoid culture. Cryopreserved epithelial crypts isolated from pig and equine jejunum were successfully used after thawing for the culture of organoids [25, 27]. Intestinal tissue fragments can also be cryopreserved and used to culture organoids as shown for human biopsies [28]. The principle is to freeze small pieces of intestinal tissue before the crypt isolation step. Once stored in liquid nitrogen, these specimens can be shipped frozen, stored and later thawed to isolate crypts and generate cultures of intestinal organoids, even if a delay in organoid formation is observed. Organoids can thus be generated from biobanked tissues following the best lab schedule. In addition, in our hands, cryopreservation of tissues has guaranteed organoid culture generation free from bacterial and fungal contaminations compared to freshly generated organoids. We have successfully produced pig intestinal organoids after isolation of epithelial crypts from small pieces (< 1 mm^2^) of several digestive segments cryopreserved in FBS (90%) and dimethyl sulfoxide (10%) (Blanc et al., unpublished data) (Figure 3, Additional file 1).

## Phenotype of livestock intestinal organoids

### Insights from transcriptome analysis

The objective of intestinal organoid cultures is to replicate in vitro as closely as possible the phenotype of the intestinal epithelium observed in vivo. Transcriptome analysis revealed that pig jejunum organoids resembled more pig jejunum tissue than the pig IPEC-J2 cell line [5]. For instance, genes coding for enterohormones or mucins were similarly expressed in jejunum tissue and organoids while they were not expressed in IPEC-J2, probably because of the lack of cellular diversity in this cell line [5]. Gene expression patterns also largely overlapped between bovine epithelial crypts and enteroids [22]. Thus, livestock intestinal organoids appear to efficiently reproduce epithelial transcriptome in vitro. The ability of intestinal organoids to retain digestive-segment specific epithelial phenotype is an important feature previously observed in mice [3]. Pig organoids from jejunum expressed higher levels of genes involved in digestion and nutrient transport than organoids derived from the ileum, while the opposite was observed for immune-related genes [5]. These digestive-segment specific gene expression profiles in pig organoids are consistent with known roles of the jejunum for nutrient absorption and of the ileum for host-microbiota interaction. Another study in pig revealed specific transcription patterns in organoids derived from duodenum, jejunum and colon; although differentially expressed genes were not detailed [29]. Stability of gene expression in organoids across extended period of time and multiple passage is necessary to ensure reproducibility of the results obtained. Transcriptome analysis of pig jejunum organoid lines revealed stable gene expression profile after a long period of culture in vitro (12 weeks/17 passages) [5]. Bovine enteroid transcriptome also showed an overall stability across 5 passages [22]. Finally, inter-individual differences persisted after multiple passages of organoids obtained from two piglets [5]. This observation suggests that experiments on intestinal organoids should be performed in parallel with several organoid lines obtained from different animals in order to cover inter-individual variability. A study showed that the age-dependent expression of genes used as markers of intestinal maturation (e.g. lactase and sucrase-isomaltase) were not reproduced in jejunum enteroids generated from piglets at several developmental stages (embryonic, suckling and post-weaning) [30]. These results suggest that enteroids do not retain the epithelial phenotype associated with the age of the animal used for crypt isolation. More studies are needed to confirm this result with other digestive segments, other markers of developmental stage (e.g. related to innate immunity) and by testing different culture conditions. Future experiments using single-cell RNA sequencing will also be helpful to further characterize each epithelial cell type present in livestock intestinal organoids.

### Epithelial polarization

**Figure 4:**
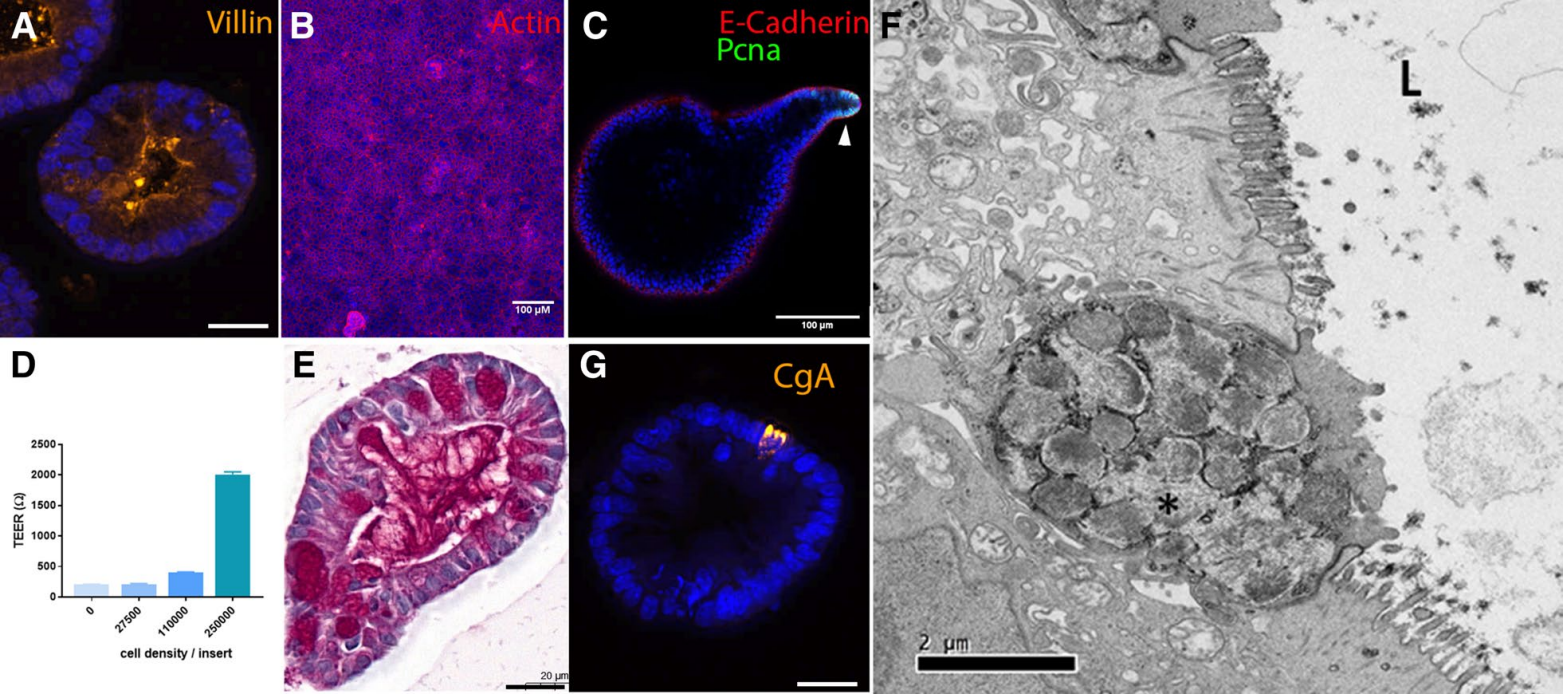
**Characterization of intestinal organoids from farm animals**. **A** Pig colon organoids stained for Villin (orange). Scale bar: 20 µm. **B** Monolayer of rabbit caecum organoid cells stained for actin (red). Scale bar: 100 µm. **C** Pig colon organoid stained for E-cadherin (red), and proliferating cell nuclear antigen (PCNA, green). The arrow indicates a proliferative zone in the organoid bud. Scale bar: 100 µm. **D** Pig colon organoid cells were seeded in Transwell inserts at several densities. Transepithelial electrical resistance (TEER) of pig organoid cell monolayers was measured 3 days post-seeding. Kruskal–Wallis test indicated a significant effect of cell density on TEER. **E** Pig colon organoid stained for mucins (Periodic Acid-Schiff staining). Scale bar: 20 µm. **F** Characterization of chicken intestinal organoid by transmission electron microscopy. The polarized organization of the cells, the brush border and the intracellular dense vesicles containing packaged mucins (marked with asterisk) were morphologically distinguishable. L: lumen. Scale bar: 2 µm. **G** Pig colon organoid stained for chromogranin A (CgA, orange). Scale bar: 20 µm. **A**, **B**, **C** and **G**: DNA (nuclei) is stained in blue.

**Figure 5:**
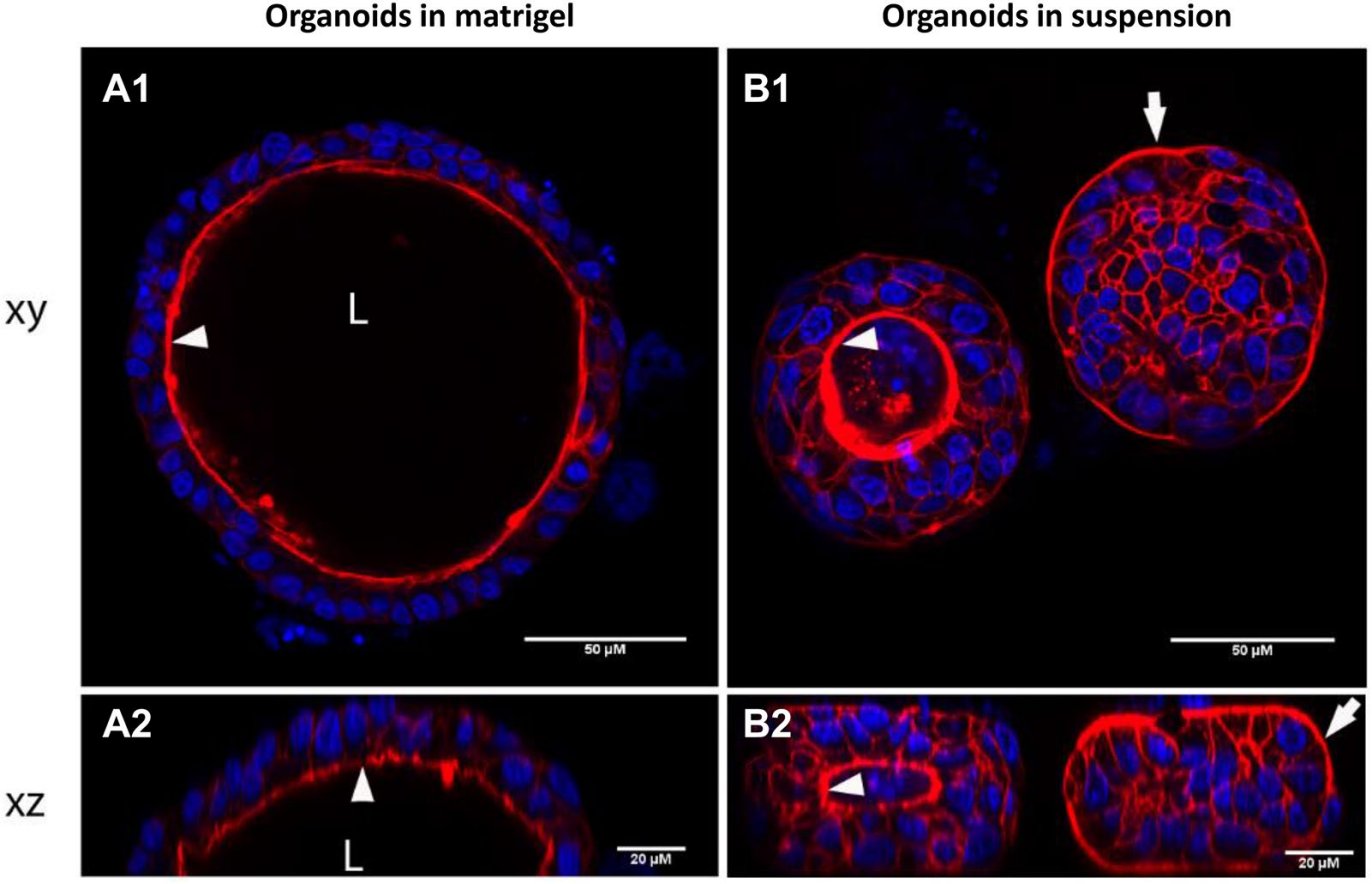
**Reversal of epithelial polarity in piglet colon organoid. **After 7 days of culture in Matrigel™ (**A**), piglet colon organoids were cultured in suspension for 24 h (**B**). Organoids were observed by confocal laser scanning microscopy to visualize horizontal (xy, A1 and B1) and vertical (xz, A2 and B2) sections. Phalloidin staining (red) shows actin and DAPI staining (blue) shows nuclei. Arrows indicate the apical side of epithelial cells. *L* Lumen.

**Figure 6:**
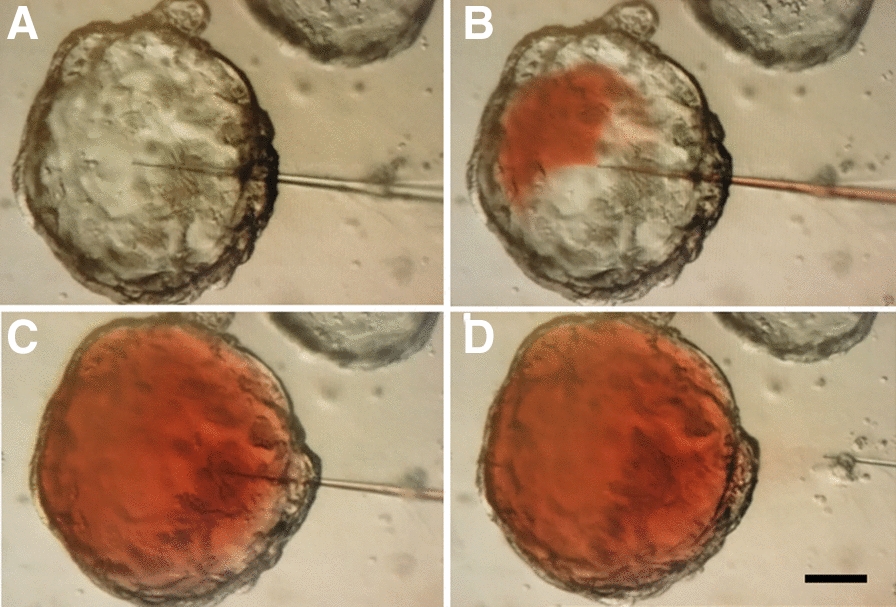
**Microinjection in a pig colon organoid to access the luminal side: initial experiments using a non-toxic food dye**. The micro-injection was monitored under a microscope and successively shows the insertion of the needle into the organoid (**A** and **B**), the injection of the dye into the pig organoid (**B** and **C**) and the removal of the needle (**D**). Organoid size: 200 µm.

IEC are highly polarized and interactions with luminal components (nutrients, microorganisms or toxins) occur at their apical side. The localization of apical surface markers (actin or villin) or visualization of microvilli by transmission electronic microscopy at the luminal side indicates that epithelial cells are also polarized in vitro in intestinal organoids from pig, cow, chicken and rabbit [16, 20, 22, 25, 31] (Figures 4A, F and 5A). Although physiologically relevant, this 3D organization leads to a basolateral exposure of experimental treatments added in the culture medium. Thus, the apical side of organoid epithelial cells is not directly accessible. The microinjection technique was used to inject micro-organisms into organoid lumen [32]. This procedure is labor-intensive and to our knowledge, no study using micro-injection in livestock intestinal organoids has been published yet. Moreover, it requires a hollow structure in the organoid (lumen) that is often reduced in differentiated organoids. We have successfully used this technique with pig colon organoids (Cherbuy et al., unpublished data) (Figure 6). However, concerns have been raised regarding reproducibility of this technique due to the variable volume injected in each organoid and unintended leak into the medium. A recent study showed that human enteroid cell polarity can be reversed by removal of the extracellular matrix [33]. The method developed by Co et al. was applied to reverse epithelial polarity in pig jejunum enteroids in suspension cultures [34]. However, in this study, the polarity failed to be reversed in ~20% organoids after 3 days, which indicates phenotypic variability in these culture conditions. We also have observed that the transfer of pig colon organoids from Matrigel™ to suspension culture for 24 h induces epithelial polarity reversal in some but not all organoids (Figure 5B, additional file 1) (Beaumont et al., unpublished data). This polarity inversion was associated with the loss of the organoid hollow structure (lumen), as observed previously in human enteroids [33]. In our hands, we observed a rapid viability loss of organoids cultured in suspension (< 3 days) which restricts the use of this culture condition to short-term experimental treatments. Alternatively, incubation of human intestinal organoids with an inflammatory cocktail composed of tumor necrosis factor-alpha, interleukin-6 and interleukin-1 induced the inversion of organoid polarity [35]. This technique has not been applied yet to reverse epithelial polarity in farm animal organoids.

The culture of 2D monolayers of organoid epithelial cells is another method to facilitate the access to the apical side and it has been applied to pig, rabbit and bovine intestinal organoids [16, 19, 21, 23, 36–38] (Figure 4B). To our knowledge, this model has not been developed in chicken and other farm animals yet. The principle is to seed dissociated intestinal organoid cells in Transwells inserts or 96-well plates previously incubated with diluted Matrigel™ (0.5–2.5% v/v) or collagen. This coating procedure produces a thin layer of extracellular matrix, which allows the attachment and growth of organoid epithelial cells in 2D but not in 3D. The culture of pig, rabbit and bovine intestinal organoid cells in Transwell inserts produced a tight monolayer and the increased transepithelial electrical resistance (TEER) overtime indicated the gradual formation of an intact epithelial barrier [19, 23]. In this 2D setting, the apical side of organoid epithelial cells faces up and is thus accessible to experimental treatments [16, 19, 37]. Epithelial differentiation can be induced in 2D monolayer by removing niche factors (as described above) or by using an air liquid interface, as shown for pig enteroids [37]. In the latter case, pig organoid cell monolayers were cultured on Transwells for two days in proliferation medium before establishment of the air liquid interface on the third day by removal of the medium in the upper (apical) compartment combined with the switch to differentiation medium lacking Wnt in the lower (basal) compartment [37]. The main limitation of monolayers of epithelial cells is the loss of the complex 3D organization of intestinal organoids such as buddings that reproduces in vitro crypt domains.

### Barrier function

A crucial function of the intestinal epithelium is to form a physical and immunological barrier to prevent the entry in the organism of harmful luminal components. The constant renewal of IEC is an important mechanism to maintain epithelial integrity. Stem/progenitor cells and proliferation markers (LGR5, SOX9, KI67, PCNA) are expressed in pig, rabbit, horse, chicken and cow organoids, which indicates that epithelial proliferation is maintained in vitro [14–16, 19, 21–23, 25, 27, 39–41] (Figure 4C). Epithelial permeability is controlled by tight and adherens junction proteins (TJP1, OCLN, CDH1) that are expressed in pig, rabbit and bovine intestinal organoids [5, 16, 22, 23, 42]. TEER measurement and analysis of apical-to-basal transport of fluorescent probes indicated that 2D monolayer of pig, rabbit and bovine organoid cells is an efficient model to study paracellular epithelial permeability [16, 19, 23] (Figure 4D, Additional file 1). Mucin 2, the main gel-forming mucin secreted by goblet cells, is expressed by intestinal organoids from pig, rabbit, horse and cows [16, 19–23, 25, 27, 43] (Figure  4E and F). Expression of antimicrobial peptides or Paneth cell markers (REG3G, LYZ) was also detected in pig, horse, cow and rabbit organoids [16, 19, 22, 25, 39]. Overall, the main cellular and molecular components required to study the epithelial barrier function are reproduced in vitro in livestock intestinal organoids. For instance, experiments in pig intestinal organoids showed that the food contaminant mycotoxin deoxynivalenol impairs epithelial renewal [18]. Another study in pig enteroids showed that in vitro heat stress (42 °C versus 37 °C) reduced the expression of proliferation markers and tight junction proteins in pig enteroids [26].

### Nutrient absorption

Besides its role of barrier, the intestinal epithelium also contributes to food digestion, nutrient absorption and hormonal regulation. Markers of absorptive enterocytes (ALPI, KRT20), digestive enzymes (sucrase-isomaltase), ion/nutrient transporters (MCT1, SGLT1, NHE3) are expressed with the appropriate cellular localization in pig, rabbit, chicken and cow organoids [15, 16, 19–21, 37, 39, 41]. Moreover, the characteristic microvilli of mature enterocytes were observed in pig and chicken enteroids [19, 40] (Figure 4F). Markers of enteroendocrine cells (PYY, CHGA) that are involved in digestive hormone secretion are also expressed in pig, rabbit and bovine organoids [16, 19–21, 23, 25, 27, 39] (Figure 4G). Thus, farm animal intestinal organoids have a great potential to study nutrient transport and gut hormone production, as it has already been performed in mouse or human enteroids [33, 44]. Experiments in 2D monolayers of pig organoid cells demonstrated that nutrients (amino acids, vitamins and choline) were transported from the apical to the basolateral side of epithelial cells, indicating functional nutrient absorption [5]. Intestinal organoids can also be used to test the effects of nutrients on epithelial homeostasis. For instance, the treatment of piglet organoids with dietary vitamin A reduced buddings and markers for differentiated cells while it increased stem cell markers [45]. In another study, treatment of pig organoids with glutamate increased epithelial proliferation [46].

## Host-microbe interactions in livestock intestinal organoids

### Enteric infections modeling

Intestinal 3D and 2D organoid models can be used to characterize the effects of enteric pathogens on the epithelium, providing valuable insights into host–microbe interactions. Indeed, the different steps of the infection can be modelled. For example, the mechanisms of entry (on the basolateral or apical side), intracellular replication and propagation but also the exit of pathogens can be dissected with the help of organoids. More particularly, the role of specific cell types in these processes or the impact of the infection on the intestinal epithelium development and cell differentiation was impossible to study in vitro prior to the development of organoids. From now on, the inflammatory responses induced by pathogens may be reproduced in vitro thanks to the organoid model. For example, studies using intestinal organoids infected with pathogens may give insights into the effects of microbial antigens on the function of Paneth cells, leading to the release of antimicrobial factors into the gut lumen.

Livestock intestinal organoids were successfully infected by a variety of enteric pathogens. Pig intestinal organoids were infected by several swine coronaviruses (porcine epidemic diarrhea virus (PEDV), transmissible gastroenteritis virus (TGEV) and porcine deltacoronavirus (PDCoV)) [21, 29, 34, 38], by the bacteria *Salmonella* Typhimurium [20] and *Lawsonia intracellularis* [36] and by the protozoan parasite *Toxoplasma gondii* [20]. Bovine intestinal organoids were infected by *Salmonella* Typhimurium*, Toxoplasma gondii* and by group A rotaviruses [20, 24]. To study the infection process, it is essential to replicate the physiological route of entry of enteric pathogen. Apical-out organoids and 2D organoid cell monolayers can be used to study enteric pathogens that penetrate epithelial cells through the apical side, as performed for the study of swine coronaviruses or *Lawsonia intracellularis* in pig intestinal organoids [21, 29, 34, 36, 38]. Alternatively, the apical surface of organoid epithelial cells can be exposed to luminal components when organoids are mechanically disrupted during the subculture process, as performed for *Toxoplasma gondii* or *Salmonella* Typhimurium infection in pig and bovine intestinal organoids [20]. However, both apical and basolateral sides of organoid cells are exposed in this setting, which might not be physiologically relevant.

The multicellular composition of intestinal organoids enables the identification of epithelial cell types targeted by enteric pathogens. For instance, double immunostaining experiments in pig enteroids revealed that PEDV and PDCoV infected mainly enterocytes, stem cells and goblet cells [21, 29, 38]. Intestinal organoids can also be used to unravel the digestive-segment tropism of enteric pathogens. Indeed, intestinal organoids retain in vitro the phenotype of their digestive segment of origin, as discussed above for pig enteroids [5, 29]. The susceptibility to PEDV and PDCoV infection was higher in pig small intestine organoids when compared to colon organoids, reflecting in vivo observations [21, 29]. In the case of PDCoV, the jejunum tropism was associated with a higher expression of the PDCoV entry receptor aminopeptidase N in jejunum enteroids, when compared to organoids derived from other digestive segments [29]. Intestinal organoids can also be used to study epithelial innate immune responses triggered by enteric pathogens. For instance, swine coronaviruses (PEDV, PDCoV and TGEV) regulated the gene expression of type-I interferons and inflammatory cytokines [21, 29, 38]. Finally, intestinal organoids are suitable to study the effects of toxins produced by enteric pathogens. For instance, the treatment of bovine ileal enteroids with Shiga toxin-containing supernatant from *Escherichia coli* reduced organoid growth [47].

### Microbiota-epithelium interactions

In vivo, the gut epithelium is in constant contact with a vast array of commensal microorganisms, collectively called the gut microbiota. Intestinal epithelial cells play a major role in maintaining the balance between the tolerance of commensal microbes and defense against them [1]. In turn, the gut microbiota regulates the main functions of the intestinal epithelium (i.e. digestion and nutrient absorption, barrier function). In recent studies, intestinal organoids have been used as accurate in vitro models to further decipher the complex interplay between individual resident microorganisms and the epithelium. For instance, studies using murine intestinal organoids revealed a link between gut microbiota and epithelial regeneration. The underlying mechanisms involve a constituent of bacterial cell walls [48] and commensal bacteria-derived short-chain fatty acids [49]. In another study, mouse ileal organoids were exposed to two specific commensal gut bacteria *Akkermansia muciniphila* and *Faecalibacterium prausnitzii* or to bacterial metabolites [50]. This study revealed a modulation in the expression of genes involved in host lipid metabolism and epigenetic activation/silencing of gene transcription [50]. Besides monoculture of bacteria with organoids, a complex human gut microbiota was cultivated for 4 days after microinjection in colon organoids [51].

Only few studies in livestock intestinal organoids have started exploring microbiota-epithelium interactions. The probiotic strain *Lactobacillus acidophilus* and the TLR2 ligand Pam3CSK4 promoted the growth of chicken embryo intestinal epithelial organoids [31]. A recent study has investigated the impact of gut microbiota-derived metabolites on rabbit cecum organoids [52]. Sterile supernatant of caecal contents prepared from suckling rabbits ingesting or not solid foods were incubated with rabbit caecal organoids. Data shows that the changes in the luminal environment (i.e*.* metabolites) at the suckling-to-weaning transition regulate gene expression in epithelial cells, and can contribute to the maturation of the gut barrier [52]. Future developments are needed to colonize livestock intestinal organoids with monoculture of commensal bacteria or with a complex microbiota. The culture of intestinal organoids from germ-free farm animals would also highlight the role of the gut microbiota on epithelial physiology. In the coming years, intestinal organoids will be helpful to understand the role of the gut microbiota in the regulation of key digestive functions such as nutrient absorption and resistance to enteric infections in farm animals.

## Future directions of research with livestock intestinal organoids

Since organoids develop from stem cells, they provide an ideal source of material for genome editing strategies, producing transgenic cells ready to clonally expand and differentiate. In genotype-to-phenotype research in farm animals, organoids make powerful systems for testing candidate causal mutations in intestinal epithelial cells [53]. Genome editing in livestock intestinal organoids also has a great potential to explore molecular mechanisms, such as the identification of receptors involved in enteric pathogen invasion. Pig intestinal organoids have been successfully transduced with lentivirus [25]. Organoids are also amenable to gene editing by CRISPR-Cas9 technologies but, to our knowledge, it has not been used in farm animal intestinal organoids yet. Alternatively, enteroids obtained from genetically edited animals can be used to study intestinal epithelial biology. For instance, enteroids were produced from pigs genetically edited to express a mutation in the *MYO5B* gene, which is involved in microvillus inclusion disease in humans [37]. *MYO5B* mutant pig enteroids displayed a poorly developed brush border and an abnormal localization of several transporters, these epithelial phenotypes being also observed in vivo in mutant pigs and in human patients [37]. Overall, farm animal intestinal organoids represent a powerful in vitro tool to study the impact of genome modifications on epithelial physiology.

Livestock intestinal organoids derived from tissue-IESC are composed only of epithelial cells. This feature is a clear advantage to study direct effects of experimental treatments on epithelial cells. However, the complexity of the intestinal mucosa is not reproduced in these organoids obtained from tissue-derived IESC. Immune, neuronal, mesenchyme and vascular cells are lacking. Oxygen gradients, mechanical forces (peristalsis motions and shear stress) and the microbiota are also not reproduced in current models of farm animal intestinal organoids despite all these factors are key regulators of intestinal physiology. In order to increase the complexity of livestock intestinal organoids, efforts should be made to develop: (i) co-culture models (e.g. epithelial organoids and immune cells), (ii) iPS/ES-derived organoid culture containing mesenchymal cells, (iii) 3D-bioprinting of epithelial and non-epithelial cells and (iv) microfluidic intestine-on-a-chip models. The latter devices were initially developed with cell lines such as Caco-2 [54] and recently with gut organoid [55, 56]. In these models, organoid dissociated cells are cultured on a porous extracellular matrix-coated membrane within a microfluidic device under flow. This allows multi-lineage differentiation and the formation of epithelium architecture similar to that of intestinal organoids but with normal cell–cell and cell-lumen interfaces and mimicking the complex physical and biochemical microenvironment. To our knowledge, intestine-on-a-chip technology has not been applied to farm animals.

Experiments performed in livestock intestinal organoids can provide ground data for less- and/or better-defined experiments and validations of hypotheses in vivo. This has a strong ethical benefit for reducing the number of animals used in vivo (3R rule: reduce, refine and replace). The creation of biobanks for farm animal intestinal organoids available as open-resources to the research community would also greatly contribute to the reduction of experiments in live animals. As a pre-requisite for the creation of shared-biobanks, harmonization of protocols used for the culture of livestock intestinal organoid (e.g. culture media composition) and the proposal of guidelines to ensure the reproducibility of models across laboratories would be required. Indeed, methods to culture intestinal organoids from livestock animals are clearly not unified yet (Tables 1, 2 and 3). So far, the protocols used to culture farm animal intestinal organoids have been directly inspired from those developed for mice or humans. It may be judicious to take into account the specificities related to the intestinal physiology of each of the animal species studied (e.g. in relation with the presence or absence of Paneth cells). It is also crucial to fully characterize the status of animals from which IESC are isolated to produced intestinal organoids (e.g. developmental stage, diet, gut microbiota, infectious status) since IESC imprinting in vivo might impact long term epithelial phenotype in organoids in vitro [57, 58]. However, human studies showed that traces of epigenetic regulation in intestinal organoids (related for example to effects of microbiota or chronic disease condition) were progressively erased during cell culture passaging for amplification [59]. Study of epigenetic regulations in livestock intestinal organoids is another important field that has not been explored yet.

In many areas, organoids are replacing all traditional in vitro models. In livestock, the development of this biological tool is all the more important as it represents the only cell models available for certain species. It is important to note that intestinal organoid models have not been developed for all farm animals yet. For instance, the development of fish intestinal organoids could clearly benefit the fish farming sector. The fields of potential application of intestinal organoids in livestock are multiple (Figure 1C) ranging from studies of phenotyping, genome editing, screening of molecules/microbes or basic science in relation to production traits such as resilience, feed efficiency, and susceptibility/resistance to disease. Most of these applications rely on the possibility of high throughput screening. It is therefore necessary to look for the optimal conditions to carry out these tests based on organoids, for example in terms of homogeneity between culture wells, sufficient cell production (e.g. large number are required for monolayer cultures) and cost-effective solutions.

## Conclusions

In this review, we described the current state of the art of the emerging field of intestinal organoids obtained from tissue-derived IESC in livestock species. Culture methods are now available to generate intestinal organoids from the main farm animals (pig, bovine, horse, sheep, rabbit and chicken). Like their mouse and human counterparts, farm animal intestinal organoids retain characteristics of their digestive segment of origin, contain all epithelial cell types and recapitulate the main epithelial functions. Most of the studies with farm animal intestinal organoids aimed to model enteric infections but numerous other applications are foreseen in veterinary, agricultural and biomedical sciences. Further developments of livestock intestinal organoids are still required to facilitate the access to the lumen, to increase cellular complexity and to reproduce essential features of the gut environment, including the presence of the microbiota. Creation of shared biobanks of cryopreserved farm animal intestinal organoids and harmonization of culture conditions will be needed to increase accessibility and reproducibility of these innovative in vitro culture systems of the intestinal epithelium. Finally, the development of livestock intestinal organoids represents a great opportunity to reduce efficiently the number of animals used for in vivo experiments.

## Supplementary Information

**Additional file 1:** **Additional material and methods**.

## Abbreviations

BMP: Bone morphogenic protein
2D: 2-Dimensional
3D: 3-Dimensional
DMSO: Dimethylsulfoxyde
DTT: Dithiothreitol
EDTA: Ethylenediaminetetraacetic acid
EGF: Epidermal growth factor
ES: Embryonic stem cell
FBS: Fetal bovine serum
IEC: Intestinal epithelial cell
IESC: Intestinal epithelial stem cell
iPS: Induced pluripotent stem
MAPK: Mitogen-activated protein kinase
PDCoV: Porcine deltacoronavirus
PEDV: Porcine epidemic diarrhea virus
TEER: Transepithelial electrical resistance
TGEV: Transmissible gastroenteritis virus
TGF-β: Transforming Growth Factor-Beta
TNF-α: Tumor necrosis factor-alpha

## References

[1] Peterson LW, Artis D (2014). Intestinal epithelial cells: regulators of barrier function and immune homeostasis. Nat Rev Immunol.

[2] Gehart H, Clevers H (2019). Tales from the crypt: new insights into intestinal stem cells. Nat Rev Gastroenterol Hepatol.

[3] Middendorp S, Schneeberger K, Wiegerinck CL, Mokry M, Akkerman RD, van Wijngaarden S, Clevers H, Nieuwenhuis EE (2014). Adult stem cells in the small intestine are intrinsically programmed with their location-specific function. Stem Cells.

[4] Vergauwen H (2015) The IPEC-J2 Cell Line. In: Verhoeckx K, Cotter P, Lopez-Exposito I et al. (eds) The Impact of Food Bioactives on Health: in vitro and ex vivo models. Cham (CH), pp 125–134. doi:10.1007/978-3-319-16104-4_12 29787039

[5] van der Hee B, Madsen O, Vervoort J, Smidt H, Wells JM (2020). Congruence of transcription programs in adult stem cell-derived jejunum organoids and original tissue during long-term culture. Front Cell Dev Biol.

[6] Randall KJ, Turton J, Foster JR (2011). Explant culture of gastrointestinal tissue: a review of methods and applications. Cell Biol Toxicol.

[7] Sato T, Vries RG, Snippert HJ, van de Wetering M, Barker N, Stange DE, van Es JH, Abo A, Kujala P, Peters PJ, Clevers H (2009). Single Lgr5 stem cells build crypt-villus structures in vitro without a mesenchymal niche. Nature.

[8] M Stelzner M Helmrath JC Dunn SJ Henning CW Houchen C Kuo J Lynch L Li ST Magness MG Martin MH Wong J Yu Consortium NIHISC 2012 A nomenclature for intestinal in vitro cultures Am J Physiol Gastrointest Liver Physiol 302 G1359 1363 10.1152/ajpgi.00493.2011 10.1152/ajpgi.00493.2011 PMC3378093 22461030

[9] Sato T, Stange DE, Ferrante M, Vries RG, Van Es JH, Van den Brink S, Van Houdt WJ, Pronk A, Van Gorp J, Siersema PD, Clevers H (2011). Long-term expansion of epithelial organoids from human colon, adenoma, adenocarcinoma, and Barrett's epithelium. Gastroenterology.

[10] In JG, Foulke-Abel J, Estes MK, Zachos NC, Kovbasnjuk O, Donowitz M (2016). Human mini-guts: new insights into intestinal physiology and host-pathogen interactions. Nat Rev Gastroenterol Hepatol.

[11] Gao X, Nowak-Imialek M, Chen X, Chen D, Herrmann D, Ruan D, Chen ACH, Eckersley-Maslin MA, Ahmad S, Lee YL, Kobayashi T, Ryan D, Zhong J, Zhu J, Wu J, Lan G, Petkov S, Yang J, Antunes L, Campos LS, Fu B, Wang S, Yong Y, Wang X, Xue SG, Ge L, Liu Z, Huang Y, Nie T, Li P, Wu D, Pei D, Zhang Y, Lu L, Yang F, Kimber SJ, Reik W, Zou X, Shang Z, Lai L, Surani A, Tam PPL, Ahmed A, Yeung WSB, Teichmann SA, Niemann H, Liu P (2019). Establishment of porcine and human expanded potential stem cells. Nat Cell Biol.

[12] Bogliotti YS, Wu J, Vilarino M, Okamura D, Soto DA, Zhong C, Sakurai M, Sampaio RV, Suzuki K, Izpisua Belmonte JC, Ross PJ (2018). Efficient derivation of stable primed pluripotent embryonic stem cells from bovine blastocysts. Proc Natl Acad Sci U S A.

[13] Sato T, Clevers H (2013). Growing self-organizing mini-guts from a single intestinal stem cell: mechanism and applications. Science.

[14] Powell RH, Behnke MS (2017). WRN conditioned media is sufficient for in vitro propagation of intestinal organoids from large farm and small companion animals. Biol Open.

[15] Panek M, Grabacka M, Pierzchalska M (2018). The formation of intestinal organoids in a hanging drop culture. Cytotechnology.

[16] Mussard E, Pouzet C, Helies V, Pascal G, Fourre S, Cherbuy C, Rubio A, Vergnolle N, Combes S, Beaumont M (2020). Culture of rabbit caecum organoids by reconstituting the intestinal stem cell niche in vitro with pharmacological inhibitors or L-WRN conditioned medium. Stem Cell Res.

[17] Holmberg FE, Seidelin JB, Yin X, Mead BE, Tong Z, Li Y, Karp JM, Nielsen OH (2017). Culturing human intestinal stem cells for regenerative applications in the treatment of inflammatory bowel disease. EMBO Mol Med.

[18] Li XG, Zhu M, Chen MX, Fan HB, Fu HL, Zhou JY, Zhai ZY, Gao CQ, Yan HC, Wang XQ (2019). Acute exposure to deoxynivalenol inhibits porcine enteroid activity via suppression of the Wnt/beta-catenin pathway. Toxicol Lett.

[19] van der Hee B, Loonen LMP, Taverne N, Taverne-Thiele JJ, Smidt H, Wells JM (2018). Optimized procedures for generating an enhanced, near physiological 2D culture system from porcine intestinal organoids. Stem Cell Res.

[20] Derricott H, Luu L, Fong WY, Hartley CS, Johnston LJ, Armstrong SD, Randle N, Duckworth CA, Campbell BJ, Wastling JM, Coombes JL (2019). Developing a 3D intestinal epithelium model for livestock species. Cell Tissue Res.

[21] Li L, Fu F, Guo S, Wang H, He X, Xue M, Yin L, Feng L, Liu P (2019). Porcine intestinal enteroids: a new model for studying enteric coronavirus porcine epidemic diarrhea virus infection and the host innate response. J Virol.

[22] Hamilton CA, Young R, Jayaraman S, Sehgal A, Paxton E, Thomson S, Katzer F, Hope J, Innes E, Morrison LJ, Mabbott NA (2018). Development of in vitro enteroids derived from bovine small intestinal crypts. Vet Res.

[23] Topfer E, Pasotti A, Telopoulou A, Italiani P, Boraschi D, Ewart MA, Wilde C (2019). Bovine colon organoids: from 3D bioprinting to cryopreserved multi-well screening platforms. Toxicol In Vitro.

[24] Alfajaro MM, Kim JY, Barbe L, Cho EH, Park JG, Soliman M, Baek YB, Kang MI, Kim SH, Kim GJ, Park SI, Pendu JL, Cho KO (2019). Dual recognition of sialic acid and alphaGal epitopes by the VP8* domains of the bovine rotavirus G6P[5] WC3 and of its mono-reassortant G4P[5] RotaTeq vaccine strains. J Virol.

[25] Khalil HA, Lei NY, Brinkley G, Scott A, Wang J, Kar UK, Jabaji ZB, Lewis M, Martin MG, Dunn JC, Stelzner MG (2016). A novel culture system for adult porcine intestinal crypts. Cell Tissue Res.

[26] Zhou JY, Huang DG, Zhu M, Gao CQ, Yan HC, Li XG, Wang XQ (2020). Wnt/beta-catenin-mediated heat exposure inhibits intestinal epithelial cell proliferation and stem cell expansion through endoplasmic reticulum stress. J Cell Physiol.

[27] Stewart AS, Freund JM, Gonzalez LM (2018). Advanced three-dimensional culture of equine intestinal epithelial stem cells. Equine Vet J.

[28] Tsai YH, Czerwinski M, Wu A, Dame MK, Attili D, Hill E, Colacino JA, Nowacki LM, Shroyer NF, Higgins PDR, Kao JY, Spence JR (2018). A method for cryogenic preservation of human biopsy specimens and subsequent organoid culture. Cell Mol Gastroenterol Hepatol.

[29] Yin L, Chen J, Li L, Guo S, Xue M, Zhang J, Liu X, Feng L, Liu P (2020). Aminopeptidase N expression, not interferon responses, determines the intestinal segmental tropism of porcine deltacoronavirus. J Virol.

[30] Mohammad MA, Didelija IC, Stoll B, Burrin DG, Marini JC (2020). Modeling age-dependent developmental changes in the expression of genes involved in citrulline synthesis using pig enteroids. Physiol Rep.

[31] Pierzchalska M, Panek M, Czyrnek M, Gielicz A, Mickowska B, Grabacka M (2017). Probiotic Lactobacillus acidophilus bacteria or synthetic TLR2 agonist boost the growth of chicken embryo intestinal organoids in cultures comprising epithelial cells and myofibroblasts. Comp Immunol Microbiol Infect Dis.

[32] Heo I, Dutta D, Schaefer DA, Iakobachvili N, Artegiani B, Sachs N, Boonekamp KE, Bowden G, Hendrickx APA, Willems RJL, Peters PJ, Riggs MW, O’Connor R, Clevers H (2018). Modelling *Cryptosporidium *infection in human small intestinal and lung organoids. Nat Microbiol.

[33] Co JY, Margalef-Catala M, Li X, Mah AT, Kuo CJ, Monack DM, Amieva MR (2019). Controlling epithelial polarity: a human enteroid model for host-pathogen interactions. Cell Rep.

[34] Li Y, Yang N, Chen J, Huang X, Zhang N, Yang S, Liu G, Liu G (2020). Next-generation porcine intestinal organoids: an apical-out organoid model for swine enteric virus infection and immune response investigations. J Virol.

[35] d'Aldebert E, Quaranta M, Sebert M, Bonnet D, Kirzin S, Portier G, Duffas JP, Chabot S, Lluel P, Allart S, Ferrand A, Alric L, Racaud-Sultan C, Mas E, Deraison C, Vergnolle N (2020). Characterization of human colon organoids from inflammatory bowel disease patients. Front Cell Dev Biol.

[36] Resende TP, Medida RL, Vannucci FA, Saqui-Salces M, Gebhart C (2020) Evaluation of swine enteroids as in vitro models for Lawsonia intracellularis infection. J Anim Sci 98: skaa011. doi:10.1093/jas/skaa011 10.1093/jas/skaa011 PMC7007770 31943029

[37] Engevik AC, Coutts AW, Kaji I, Rodriguez P, Ongaratto F, Saqui-Salces M, Medida RL, Meyer AR, Kolobova E, Engevik MA, Williams JA, Shub MD, Carlson DF, Melkamu T, Goldenring JR (2020). Editing myosin VB gene to create porcine model of microvillus inclusion disease, with microvillus-lined inclusions and alterations in sodium transporters. Gastroenterology.

[38] Luo H, Zheng J, Chen Y, Wang T, Zhang Z, Shan Y, Xu J, Yue M, Fang W, Li X (2020). Utility evaluation of porcine enteroids as PDCoV infection model in vitro. Front Microbiol.

[39] Gonzalez LM, Williamson I, Piedrahita JA, Blikslager AT, Magness ST (2013). Cell lineage identification and stem cell culture in a porcine model for the study of intestinal epithelial regeneration. PLoS One.

[40] Li J, Li J, Zhang SY, Li RX, Lin X, Mi YL, Zhang CQ (2018). Culture and characterization of chicken small intestinal crypts. Poult Sci.

[41] Pierzchalska M, Grabacka M, Michalik M, Zyla K, Pierzchalski P (2012). Prostaglandin E2 supports growth of chicken embryo intestinal organoids in Matrigel matrix. Biotechniques.

[42] Pierzchalska M, Grabacka M (2016). The potential role of some phytochemicals in recognition of mitochondrial damage-associated molecular patterns. Mitochondrion.

[43] Ferrandis Vila M, Trudeau MP, Hung YT, Zeng Z, Urriola PE, Shurson GC, Saqui-Salces M (2018). Dietary fiber sources and non-starch polysaccharide-degrading enzymes modify mucin expression and the immune profile of the swine ileum. PLoS One.

[44] Zietek T, Rath E, Haller D, Daniel H (2015). Intestinal organoids for assessing nutrient transport, sensing and incretin secretion. Sci Rep.

[45] Wang Z, Li J, Wang Y, Wang L, Yin Y, Yin L, Yang H, Yin Y (2020). Dietary vitamin A affects growth performance, intestinal development, and functions in weaned piglets by affecting intestinal stem cells. J Anim Sci.

[46] Zhu M, Qin YC, Gao CQ, Yan HC, Wang XQ (2020). l-Glutamate drives porcine intestinal epithelial renewal by increasing stem cell activity via upregulation of the EGFR-ERK-mTORC1 pathway. Food Funct.

[47] Fitzgerald SF, Beckett AE, Palarea-Albaladejo J, McAteer S, Shaaban S, Morgan J, Ahmad NI, Young R, Mabbott NA, Morrison L, Bono JL, Gally DL, McNeilly TN (2019). Shiga toxin sub-type 2a increases the efficiency of *Escherichia coli* O157 transmission between animals and restricts epithelial regeneration in bovine enteroids. PLoS Pathog.

[48] Nigro G, Rossi R, Commere PH, Jay P, Sansonetti PJ (2014). The cytosolic bacterial peptidoglycan sensor Nod2 affords stem cell protection and links microbes to gut epithelial regeneration. Cell Host Microbe.

[49] Park JH, Kotani T, Konno T, Setiawan J, Kitamura Y, Imada S, Usui Y, Hatano N, Shinohara M, Saito Y, Murata Y, Matozaki T (2016). Promotion of intestinal epithelial cell turnover by commensal bacteria: role of short-chain fatty acids. PLoS One.

[50] Lukovac S, Belzer C, Pellis L, Keijser BJ, de Vos WM, Montijn RC, Roeselers G (2014) Differential modulation by *Akkermansia muciniphila* and *Faecalibacterium prausnitzii* of host peripheral lipid metabolism and histone acetylation in mouse gut organoids. mBio. doi:10.1128/mBio.01438-14 10.1128/mBio.01438-14 PMC4145684 25118238

[51] Williamson IA, Arnold JW, Samsa LA, Gaynor L, DiSalvo M, Cocchiaro JL, Carroll I, Azcarate-Peril MA, Rawls JF, Allbritton NL, Magness ST (2018). A high-throughput organoid microinjection platform to study gastrointestinal microbiota and luminal physiology. Cell Mol Gastroenterol Hepatol.

[52] Beaumont M, Paes C, Mussard E, Knudsen C, Cauquil L, Aymard P, Barilly C, Gabinaud B, Zemb O, Fourre S, Gautier R, Lencina C, Eutamene H, Theodorou V, Canlet C, Combes S (2020). Gut microbiota derived metabolites contribute to intestinal barrier maturation at the suckling-to-weaning transition. Gut Microbes.

[53] Giuffra E, Tuggle CK, Consortium F (2019). Functional Annotation of Animal Genomes (FAANG): current achievements and roadmap. Annu Rev Anim Biosci.

[54] Shah P, Fritz JV, Glaab E, Desai MS, Greenhalgh K, Frachet A, Niegowska M, Estes M, Jager C, Seguin-Devaux C, Zenhausern F, Wilmes P (2016). A microfluidics-based in vitro model of the gastrointestinal human-microbe interface. Nat Commun.

[55] Sidar B, Jenkins BR, Huang S, Spence JR, Walk ST, Wilking JN (2019). Long-term flow through human intestinal organoids with the gut organoid flow chip (GOFlowChip). Lab Chip.

[56] Nikolaev M, Mitrofanova O, Broguiere N, Geraldo S, Dutta D, Tabata Y, Elci B, Brandenberg N, Kolotuev I, Gjorevski N, Clevers H, Lutolf MP (2020). Homeostatic mini-intestines through scaffold-guided organoid morphogenesis. Nature.

[57] Noben M, Verstockt B, de Bruyn M, Hendriks N, Van Assche G, Vermeire S, Verfaillie C, Ferrante M (2017). Epithelial organoid cultures from patients with ulcerative colitis and Crohn's disease: a truly long-term model to study the molecular basis for inflammatory bowel disease?. Gut.

[58] Abo H, Chassaing B, Harusato A, Quiros M, Brazil JC, Ngo VL, Viennois E, Merlin D, Gewirtz AT, Nusrat A, Denning TL (2020). Erythroid differentiation regulator-1 induced by microbiota in early life drives intestinal stem cell proliferation and regeneration. Nat Commun.

[59] Kraiczy J, Nayak KM, Howell KJ, Ross A, Forbester J, Salvestrini C, Mustata R, Perkins S, Andersson-Rolf A, Leenen E, Liebert A, Vallier L, Rosenstiel PC, Stegle O, Dougan G, Heuschkel R, Koo BK, Zilbauer M (2019). DNA methylation defines regional identity of human intestinal epithelial organoids and undergoes dynamic changes during development. Gut.

[60] Koltes DA, Gabler NK (2016). Characterization of porcine intestinal enteroid cultures under a lipopolysaccharide challenge. J Anim Sci.

[61] Acharya M, Arsi K, Donoghue AM, Liyanage R, Rath NC (2020). Production and characterization of avian crypt-villus enteroids and the effect of chemicals. BMC Vet Res.

[62] Pierzchalska M, Panek M, Czyrnek M, Grabacka M (2019). The three-dimensional culture of epithelial organoids derived from embryonic chicken intestine. Methods Mol Biol.

