# Supplementary material for: Intestinal organoids in farm animals
Source: Vet Res. 2021 Feb 25;52:33. doi: 10.1186/s13567-021-00909-x (PMC7905770; doi:10.1186/s13567-021-00909-x)
Supplement: Supplementary file 1 — Additional file 1: Additional material and methods. [file 13567_2021_909_MOESM1_ESM.docx]

**Supplemental materials and methods**

**Generation of pig organoids from frozen tissues**

Pig intestinal organoids were generated from intestinal tissue of 5 months old Large White pigs. Sections of intestinal tissues (duodenum, jejunum, ileum or colon) were collected at slaughter and opened longitudinally. Colon sections were washed extensively under tap water to remove faeces. All sections were washed at least twice in ice-cold PBS and incubated on ice for 30 min in decontamination medium: PBS containing 5% of Vétédine solution (Med Vet), 0.2% fungizone (500 pg/mL amphotericin B final concentration), 50 U/mL penicillin and 50 µg/mL streptomycin and 50 µg/mL gentamycin (all from Gibco). Intestinal tissue sections were then transferred to PBS and kept on ice for up to 6 h. Samples were moved to the cell culture laboratory where, under sterile condition and working on ice, they were incubated for 30 min in decontamination medium and then washed twice in ice-cold PBS. Villi were removed by gentle scrapping with a scalpel for appropriate sections. Small sections of around 1 cm^2^ were divided into small cubes of around 1mm^3^ and transferred into cryovials containing 1 mL of fetal bovine serum (FBS, Gibco) with 10% DMSO (Sigma Aldrich). Cryovials were frozen slowly (in a freezing container with isopropanol placed at -80 °C for 24-48 h) and then transferred to liquid nitrogen for long-term storage.

For the isolation of intestinal crypts, frozen biopsies were thawed, put on ice and washed twice with ice-cold PBS containing 5% of FBS. Biopsies were then incubated for 30 min on ice with decontamination medium and then incubated for 10 min at room temperature on a rocking platform (about 40 rpm) in 10 mL PBS – 30 mM EDTA. Supernatant was removed and 10 mL PBS – 30 mM EDTA were added for a 10 min-incubation at 37 °C. Supernatant was discarded and biopsies were washed twice with ice-cold DMEM with antibiotics (50 U/mL penicillin and 50 µg/mL streptomycin, Gibco). Tissue was then disrupted by vigorous vortexing and pipetting up and down, placed on a 100 µm cell strainer filter and crushed using a syringe piston. After washing with DMEM (Gibco) with antibiotics, isolated cells were centrifuged (5 min, 4 °C, 300 *g*) and finally suspended in 100-200 µL of medium. Cell suspension was diluted in Matrigel^TM^ (Corning® Matrigel® Growth Factor Reduced Basement Membrane Matrix, Phenol Red-free, REF 356231) and 50 µL domes were plated on a 24-well culture plate with 600 µL per wells of organoid culture medium (human IntestiCult™ Organoid Growth Medium, STEMCELL Technologies, Vancouver, Canada) with antibiotics (50 U/mL penicillin and 50 µg/mL streptomycin). Medium was replaced every 2-3 days and organoids were passaged after 7-10 days of culture. Medium was supplemented with 50 µg/mL gentamycin (Gibco) until first passage and with 10 µM RHO/ROCK pathway inhibitor Y-27632 (STEMCELL, Technologies, Vancouver, Canada) after each plating.

**Polarity reversal in porcine organoids**

Pig colon organoids were cultured in Matrigel^TM^ (Corning, Cat #354234, Corning, NY, USA) with 50% L-WRN conditioned medium (CM) produced by L-WRN cells (ATCC®, CRL-3276^TM^) supplemented with 10 µM Y27632 (ATCC® ACS-3030™) and 10 µM SB-431542 (Sigma-Aldrich) as described previously [14, 16]. After 1 week, organoids were cultured in suspension (i.e. without Matrigel^TM^) based on the method developed by Co et al. [33]. Organoids were removed from Matrigel^TM^ by incubation in cold PBS (1 h, 4 °C) followed by centrifugation (50 *g*, 4 °C, 5 min). The organoid pellet was suspended in warm growth medium (50% L-WRN CM, 10 µM Y27632, 10 µM SB-431542) and seeded in 48-well plates before incubation (5% CO_2_, 37 °C). After 24 h of suspension culture, organoids were fixed with 4% paraformaldehyde (Electron Microscopy Sciences) during 20 min under agitation. Actin and DNA staining was performed as described previously [16]. In parallel, organoids grown in Matrigel^TM^ for 1 week in Nunc Lab-Tek Chamber Slide system (Thermo Fisher Scientific) were stained for actin following the same procedure.

**Porcine organoid cell monolayer**

Pig colon organoids were recovered from Matrigel^TM^, incubated in TrypLE Express (ThermoFisher) dissociation medium at 37 °C and dissociated by repeated pipetting to obtain a single cell suspension. After centrifugation, cell pellets were resuspended in 50% L-WRN CM, and counted manually using a hemocytometer. HTS Transwell 24-well culture plates (Costar) were used for TEER measurements. The membrane insert was pre-coated with 0.5% (v/v) Matrigel^TM^ in F12 medium at 37 °C for 1 h. Cells were then seeded at different densities in pre-coated culture Transwells and cultured for 3 days at 37 °C (5% CO_2_) in 50% L-WRN CM supplemented with Y27632 (10 µM). The Transepithelial electrical resistance (TEER) was assessed using the REMS Automated Tissue Resistance Measuring System (WPI). Briefly, the upper and lower electrodes were automatically inserted into each well of the microplate and the TEER was recorded. The experiment was carried out twice. The negative control used pre-coated Transwells without any cells. Statistical analyses were performed using GraphPad software. Non-parametric Kruskal-Wallis test was used to reveal difference between groups. *P*-value below 0.05 was considered significant.
